# Negative Regulation of Neuromedin U mRNA Expression in the Rat Pars Tuberalis by Melatonin

**DOI:** 10.1371/journal.pone.0067118

**Published:** 2013-07-02

**Authors:** Sayaka Aizawa, Ichiro Sakata, Mai Nagasaka, Yuriko Higaki, Takafumi Sakai

**Affiliations:** Area of Regulatory Biology, Division of Life Science, Graduate School of Science and Engineering, Saitama University, Sakuraku, Saitama, Japan; Morehouse School of Medicine, United States of America

## Abstract

The pars tuberalis (PT) is part of the anterior pituitary gland surrounding the median eminence as a thin cell layer. The characteristics of PT differ from those of the pars distalis (PD), such as cell composition and gene expression, suggesting that the PT has a unique physiological function compared to the PD. Because the PT highly expresses melatonin receptor type 1, it is considered a mediator of seasonal and/or circadian signals of melatonin. Expression of neuromedin U (NMU) that is known to regulate energy balance has been previously reported in the rat PT; however, the regulatory mechanism of NMU mRNA expression and secretion in the PT are still obscure. In this study, we examined both the diurnal change of NMU mRNA expression in the rat PT and the effects of melatonin on NMU in vivo. In situ hybridization and quantitative PCR analysis of laser microdissected PT samples revealed that NMU mRNA expression in the PT has diurnal variation that is high during the light phase and low during the dark phase. Furthermore, melatonin administration significantly suppressed NMU mRNA expression in the PT in vivo. On the other hand, 48 h fasting did not have an effect on PT-NMU mRNA expression, and the diurnal change of NMU mRNA expression was maintained. We also found the highest expression of neuromedin U receptor type 2 (NMUR2) mRNA in the third ventricle ependymal cell layer, followed by the arcuate nucleus and the spinal cord. These results suggest that NMU mRNA expression in the PT is downregulated by melatonin during the dark phase and shows diurnal change. Considering that NMU mRNA in the PT showed the highest expression level in the brain, PT-NMU may act on NMUR2 in the brain, especially in the third ventricle ependymal cell layer, with a circadian rhythm.

## Introduction

The pars tuberalis (PT), which comprises the rostral part of the anterior pituitary gland surrounding the median eminence as a thin cell layer, has characteristics that differ from those of the pars distalis (PD). In general, the mammalian PT consists of 2 cell types, folliculostellate cells and glycoprotein hormone-producing cells, i.e., thyrotropes and gonadotropes [1]. In rats, most hormone-producing cells in the PT are small, oval-shaped TSH-producing cells that are characterized by spot-like TSH immunoreactivity on the Golgi apparatus [2]. In addition, a high density of melatonin-binding sites has been observed in the PT of many species [3], [4], and melatonin receptor type 1 (MT1) is expressed in αGSU- and TSHβ-expressing cells in the rat PT [5]. Melatonin is exclusively secreted from the pineal gland during the dark period, and melatonin signal corresponds to the duration of the dark period, thereby providing photoperiodic information to the melatonin receptor-producing target sites. Hence, the PT might play an important role in the mediation of seasonal and/or circadian signals [6], [7]. In fact, the duration of the photoperiod affects the structure of TSH cells and TSHβ mRNA expression in the PT cells of the Djungarian hamster [8], [9], [10]. We have previously reported that TSHβ and αGSU mRNA expression in the rat PT has a diurnal variation, and chronic administration of melatonin suppresses TSHβ and αGSU mRNA expression and TSH secretion [11]. Furthermore, it has been suggested that TSH secreted from the PT acts on the TSH receptor of the ependymal cell layer of the mediobasal hypothalamus and regulates the expression of type 2 deiodinase, resulting in the regulation of GnRH release in seasonal animals [12], [13]. This effect of PT-TSH has also been reported in mice, which are non-seasonal breeding animals [14].

On the other hand, it is also reported that the rat PT produces neuromedin U (NMU), which is known as a gut-brain hormone [15], [16]. NMU was first isolated from the porcine spinal cord (SC) by the uterus contraction activity assay [17], [18]. NMU constitutes 25 amino acid residues in humans and 23 amino acid residues in mice and rats, each containing the highly conserved, core active C-terminal 8 amino acid residues [19], [20], [21]. The NMU knockout mouse shows hyperphagia and obesity [22], whereas the transgenic mouse overexpressing NMU is lean and hypophagic [23]. Although the precise physiological functions of NMU have yet to be clearly defined, the phenotypes of genetically modified mice provide strong support for the role of NMU in energy balance.

NMU is peripherally produced throughout the gastrointestinal tract, and it is expressed at particularly high levels in the duodenum and jejunum [24], cecum, colon, and rectum, with considerably lower levels reported in the esophagus and stomach [25]. Because of its wide distribution, a variety of peripheral activities of NMU have been reported, including regulation of smooth-muscle contractions [26], [27], local blood flow [17], [28], and ion transport in the gut [29].

In the mouse central nervous system, NMU mRNA expression is found in the suprachiasmatic (SCN), dorsomedial (DMH), ventromedial (VMH), and arcuate (ARC) nucleus of the hypothalamus [16]. In contrast, in rats, it is reported that NMU mRNA expression in these regions is weaker, and high levels of NMU mRNA expression are found in the PT [15], [16].

The NMU receptor was also found in the hypothalamus, and a specific central receptor for NMU (NMU receptor type 2 [NMUR2]) is highly expressed in the ependymal cell layer of the third ventricle and expressed at lower levels in the paraventricular nucleus (PVN) and the ARC [16], [30]. In addition, it is reported that icv injection of NMU induces an increase in c-fos protein levels in the PVN, SON, ARC, DMH, lateral hypothalamic area, and parabrachial nucleus of the brain stem in rats [15], [31], [32]. Moreover, icv injection of NMU in the rat induces various effects on feeding behavior and energy homeostasis, such as the suppression of food intake and body weight gain, and increase in gross locomotor activity, body temperature, heat production, and arterial pressure [15], [30], [31], [33], [34], [35], [36]. These studies strongly suggest that a high concentration of NMU in the cerebroventricular circulation has a potent endogenous anorexic effect through NMUR2 in the brain. However, the origin of this high concentration of NMU is still obscure [16].

Although central NMU is believed to play a physiologically important role in feeding behavior and energy homeostasis, there are only a few reports that focus on NMU in the rat PT. Furthermore, the regulatory mechanism of NMU mRNA expression in the PT is still unclear. Hence, in this study we first examined the daily change of NMU mRNA expression in the rat PT and the effects of melatonin and fasting on NMU mRNA expression, in order to clarify the regulatory mechanism of NMU mRNA expression in the rat PT.

## Materials and Methods

### Animals

Male Wistar rats weighing 200–250 g were maintained under a 12-h light/dark cycle (light switched on at 08∶00) at room temperature (23°C ±2°C) with food and water *ad libitum*. All procedures were approved and performed in accordance with the Saitama University Committee on Animal Research. All efforts were made to minimize animal suffering and to reduce the number of animals used in the experiment.

### Experimental Design

#### NMU mRNA expression regions in the brain of male Wistar rats

To determine NMU mRNA expression in the rat brain, *in situ* hybridization (ISH) on free-floating brain sections and quantitative PCR (qPCR) were performed. Rats were sacrificed under deep anesthesia with diethyl ether at Zeitgeber time (ZT) 6. Zeitgeber time is defined by dividing the 24 light/dark cycle into 24 1-hour Zeitgeber time units, with the lights-on time defined as ZT0 (08∶00) and the lights-off time defined as ZT12 (20∶00). Brains were quickly removed. For ISH on free-floating sections, brains were immersed in 4% paraformaldehyde (PFA) in 0.067 M phosphate buffer (PB), pH 7.4, for 20 h, then immersed in 30% sucrose in PBS for 48 h, and frozen with Tissue-Tek OCT compound (Sakura Finetek, Torrance, USA). For qPCR analysis, collected brains were immediately frozen in Tissue-Tek OCT compound (Sakura Finetek) without fixation, and stored at −80°C for laser microdissection (LMD).

#### NMU mRNA-expressing cell type in the PT

To analyze NMU mRNA-expressing cell types in the rat PT, we performed double staining of NMU mRNA (by fluorescent ISH) and TSH (by fluorescent immunohistochemistry [IHC]). Brains were collected at ZT6 and immersed in 4% PFA solution for 20 h, then immersed in 30% sucrose solution, and subsequently frozen in Tissue-Tek OCT compound (Sakura Finetek) for storage at −80°C until use.

#### Diurnal changes of NMU mRNA expression in the PT

To study the diurnal changes of NMU mRNA expression, rats were sacrificed under deep anesthesia with diethyl ether at ZT0, 6, 12, 18, and 24, and the brains were frozen in Tissue-Tek OCT compound (Sakura Finetek) without fixation for storage at −80°C for ISH and LMD experiments. To avoid the effects of light, the rats were decapitated with lights off during the dark phase (ZT18 and ZT24).

#### Effects of melatonin on NMU mRNA expression in the PT, PD, and SCN

To study the effects of melatonin on NMU mRNA expression, rats were divided into 2 groups: the sham-operated (sham) group and the pinealectomised and melatonin-replaced (melatonin) group. Rats in the melatonin group were chronically administered melatonin for 7 days. The anesthetized rats were fixed with a brain stereotaxis apparatus (SR-3; Narishige, Tokyo, Japan), and a hole of 2.0 mm in diameter was drilled in the lambda by using a dental drill. The pineal gland was removed using forceps. The accuracy of pinealectomy was checked visually when rats were killed. Melatonin was administered to pinealectomised rats via a 15-mm-long silastic tube (i.d., 2.0 mm; o.d., 3.0 mm; 100–2N, Kaneka Medix, Kanagawa, Japan) filled with 10 mg melatonin (Tokyo Kasei Co., Ltd, Tokyo, Japan), with the silastic tube inserted subcutaneously in the back. We have previously shown that the plasma melatonin concentration in the melatonin group of rats was approximately 227 pg/mL, which was close to the level at ZT20 in the dark phase [11]. Empty implants were inserted in sham-operated rats. Seven days after the operation, the animals were killed at ZT6 and ZT18 under deep anesthesia with diethyl ether, and their brains were quickly removed and frozen in Tissue-Tek OCT compound (Sakura Finetek) and stored at −80°C for LMD and ISH experiments.

#### Effects of fasting on NMU mRNA expression in the PT

To study the effects of fasting, rats were deprived of food for 48 h; one group was deprived of food from ZT6 (14∶00) for 48 h and the other group was deprived from ZT18 (02∶00) for 48 h. Free-feeding rats were used as controls. Brains were collected at ZT6 (14∶00) or ZT18 (02∶00), frozen in Tissue-Tek OCT compound (Sakura Finetek), and stored at −80°C for LMD.

### *In situ* Hybridization

ISH was performed as previously described [11]. In ISH on free-floating brain sections, serial 40-µm-thick frontal sections were cut and collected in PBS. Sections were washed with PBS, treated with 5 µg/mL proteinase K for 15 min, and then washed for 3 min with PBS. For ISH on fresh frozen brain sections, frozen 8-µm-thick frontal sections were cut and mounted on silane-coated slides. The sections were fixed with 4% PFA solution for 20 min, and washed for 3 min with PBS. Subsequently, sections were treated with 0.25% acetic anhydride in 0.1 M triethanolamine for 10 min and washed with PBS for 1 min for ISH on both free-floating sections and fresh frozen sections. Digoxigenin (DIG)-labeled anti-sense and sense rat NMU cRNA probes (GenBank accession No. NM_022239, position 231–625) and DIG-labeled anti-sense and sense NMU receptor 2 cRNA probes (GenBank accession No. NM_022275, position 473–1270) were synthesized using a labeling kit (Roche Diagnostics, Mannheim, Germany) with SP6 or T7 RNA polymerase (Roche Diagnostics). The probes were diluted to 1 ng/µL with hybridization buffer (50% formamide, 3× SSC, 0.12 M PB, pH 7.4, 1× Denhardt’s solution, 125 µg/mL tRNA, 0.1 mg/mL sonicated salmon sperm DNA, and 10% dextran sulfate), and placed on the tissue sections and incubated for 16 h at 60°C. The sections were subsequently immersed in 2× SSC containing 50% formamide for 30 min. The sections were then treated with TNE (10 mM Tris-HCl, pH 7.6, 500 mM NaCl, and 1 mM EDTA, pH 8.0) for 10 min and then with RNase A (1 µg/mL in TNE) for 30 min at 37°C. Next, the sections were immersed in TNE for 10 min at 37°C and washed twice with 2× SSC at 60°C for 10 min, followed by washing with 0.2× SSC for 10 min and 0.1× SSC for 10 min, twice each at 60°C. The sections were further incubated for 5 min in Buffer 1 (100 mM Tris-HCl, pH 7.5, 150 mM NaCl, and 0.01% Tween 20), immersed in 1.5% blocking reagent (Roche Diagnostics) in Buffer 1 for 1 h at 37°C, and subsequently washed in Buffer 1 for 5 min. After washing, the sections were incubated with an alkaline phosphatase-conjugated anti-DIG antibody (Roche Diagnostics) diluted 1∶1,000 in Buffer 1 for 16 h at 4°C. The sections were washed thrice in Buffer 1 for 15 min each and in Buffer 2 (100 mM Tris-HCl, pH 9.5, 100 mM NaCl, and 50 mM MgCl_2_) for 5 min. A chromagen solution (337 µg/mL 4-nitroblue tetrazolium chloride and 175 µg/mL 5-bromo-4-chloro-3-indolyl-phosphate in Buffer 2) was added, and the sections were incubated until a visible signal was detected. The reaction was stopped by adding a solution of 10 mM Tris-HCl, pH 7.6, and 1 mM EDTA, pH 8.0. The sections were washed with PBS, mounted on the glass slide, and then covered with 90% glycerol in PBS. Sections were observed under a light microscope (BX60; Olympus, Tokyo) and photographed with a digital camera (DP70; Olympus).

### Double Staining for NMU (by ISH) and TSH (by IHC)

Fixed, frozen, 5-µm-thick frontal sections were cut and mounted on silane-coated slides. Sections were washed with PBS, treated with 0.5 µg/mL proteinase K for 15 min, fixed with 4% PFA solution for 20 min, and washed for 3 min with PBS. Subsequently, sections were treated with 0.25% acetic anhydride in 0.1 M triethanolamine for 10 min and washed with PBS for 1 min. DIG-labeled anti-sense and sense rat NMU cRNA probes were diluted to 1 ng/µL with hybridization buffer, placed on the tissue sections, and incubated for 16 h at 60°C. The sections were subsequently immersed in 2× SSC containing 50% formamide for 30 min. The sections were then treated with TNE for 10 min and with RNase A (1 µg/mL in TNE) for 30 min at 37°C. Next, the sections were washed with 2× SSC for 10 min, 0.2× SSC for 10 min, and 0.1× SSC for 10 min, twice each at 60°C. The sections were further incubated for 5 min in Buffer 1, and immersed in 1.5% blocking reagent (Roche Diagnostics) in Buffer 1 for 1 h at 37°C.

After washing in Buffer 1 for 5 min, the sections were incubated overnight with the antibody for rat TSH diluted 1∶40,000 in 0.5% blocking reagent (Roche Diagnostics) in a humidified chamber for immunohistochemical detection. The production and specificity of the antibody, rabbit anti-rat TSH serum (HAC-RT29-01RBP86; a gift from the Laboratory of Biosignal Sciences, Institute for Molecular and Cellular Regulation, Gunma University), have been described elsewhere (Kawarai 1980, Wakabayashi & Tanaka 1988). The sections were incubated with both an alkaline phosphatase-conjugated anti-DIG antibody (Roche Diagnostics) diluted 1∶1,000 (for ISH) and donkey anti-rabbit IgG H&L (DyLight 488) (Abcam plc, Cambridge, UK) diluted 1∶50 in 0.5% blocking reagent (Roche Diagnostics) for 2 h at 37°C. The sections were washed thrice in PBS for 15 min each and in Buffer 3 (100 mM Tris-HCl, 100 mM NaCl, and 10 mM MgCl_2_, pH 8.0) for 5 min. The sections were incubated thrice with a filtrated HNPP/Fast Red TR mix solution (100 µg/mL 2-hydroxy-3-naphtoic acid-2′-phenylanilide phosphate and 250 µg/mL 4-chloro-2-methylbenzenediazonium hemi-zinc chloride salt) (Roche Diagnostics) for 30 min each. The reaction was stopped by washing with distilled water and then covered with 90% glycerol in PBS. Sections were observed under a fluorescence microscope (BX60; Olympus, Tokyo) and photographed with a digital camera (DP70; Olympus).

### Preparation of Sections and LMD

LMD was performed as previously described [37]. Briefly, frozen 20-µm-thick serial frontal sections were cut and mounted on MembraneSlides (cat. no. 11505189; Leica Microsystems, Wetzlar, Germany). To prevent RNA degradation during sectioning, these slides were pre-coated with 40 µL RNAlater-Ice (Ambion, Austin, USA) and stored at −20°C inside a cryostat. The sections were fixed in ice-cold acetone for 2 min, dehydrated with a graded ethanol series (75% and 50%) for 1 min each, and stained for 10 s with 0.1% toluidine blue (Sigma-Aldrich, St. Louis, USA) dissolved in 50% ethanol in RNase-free water. The sections were rinsed in RNase-free water, dehydrated in a graded ethanol series (50% and 75%) for 1 min each, immersed in 100% ethanol for 5 s, and dried using a cold air dryer for 1 min in preparation for LMD. The PT, ARC, SCN, VMH, and the ependymal cell layer (EC) were dissected from stained sections by using an LMD system (LMD 7000; Leica Microsystems), and the cut sections were directly captured into 0.2-mL tube caps filled with RLT buffer (RNeasy Micro Kit; QIAGEN, Hilden, Germany) containing β-mercaptoethanol. Total RNA was isolated using the RNeasy Micro Kit according to the manufacturer’s protocol, including on-column DNase treatment. RNase-free water (14 µL) was applied for elution. The integrity of the isolated total RNA was verified by high-resolution microcapillary electrophoresis by using the RNA 6000 Pico LabChip and the Agilent 2100 Bioanalyzer (Agilent Technologies, Waldbronn, Germany). Agilent 2100 Bioanalyzer software was used to analyze electropherograms and to quantify the 28S and 18S rRNA band intensities.

### RNA Extraction and Quantitative PCR

Total RNA from the LMD-captured PT, ARC, SCN, VMH, and EC was extracted using the RNeasy Micro Kit as described above. The PD and SC (the cervical region) were collected and immediately submerged in ISOGEN (NipponGene, Tokyo, Japan). The total RNA from the PD and SC was extracted using an ISOGEN (NipponGene) according to the manufacturer’s instructions, and it was then treated with DNase. The cDNA was synthesized from total RNA using the High Capacity RNA-to-cDNA kit (Applied Biosystems, Foster City, USA) according to the manufacturer’s instructions. The oligonucleotide-specific primers for rat NMU and NMUR2 are as follows: NMU, forward 5′-CGT TCC TCA ACT GCA TGA GA-3′, reverse 5′-CCA TTG CGT GGC CTA AAT AA-3′ (amplicon size, 105 bp) and NMUR2, forward 5′-GCG AAC AAA GTG GCT GTG AA-3′, reverse 5′-GTC CAG CAG ATG GCA AAC AC-3 ′ (amplicon size, 95 bp). The qPCR reactions were performed using a LightCycler (Roche Diagnostics) with SYBR Premix Ex Taq (TakaraBIO, Shiga, Japan). The initial template denaturation was programmed for 30 s at 95°C. PCR was performed with 40 cycles of 5 s at 95°C and 15 s at 60°C, and a final cooling step was performed for 30 s at 40°C. Rat GAPDH mRNA was used as the internal control. The expression of each mRNA is shown relative to GAPDH mRNA. All reactions were performed in duplicate, and each transcript was quantitatively measured by establishing a linear amplification curve from serial dilutions of each plasmid containing the amplicon sequence. The amplicon size and specificity were confirmed by a melting curve analysis and 2% agarose gel electrophoresis.

### Statistical Analysis

The values are given as the means ± SEM. In the experiment of diurnal changes of NMU mRNA expression in the PT, differences between each time point were evaluated by a one-way analysis of variance (ANOVA) with Tukey’s *post hoc* tests. The effect of melatonin treatment and time (ZT6 and ZT18) as well as the interaction were assessed using two-way ANOVA followed by Tukey’s test for multiple comparisons among groups, or followed by a t-test to further distinguish among the groups. In the analysis of NMUR2 mRNA expression levels, differences between ZT6 and ZT18 were evaluated by *t*-test. The statistical software used was GraphPad Prism 5 software (GraphPad Software, La Jolla, USA). Differences with *P*<0.05 were considered significant.

## Results

### NMU mRNA Expression Regions in the Brain of Male Wistar Rats

**Figure 1:**
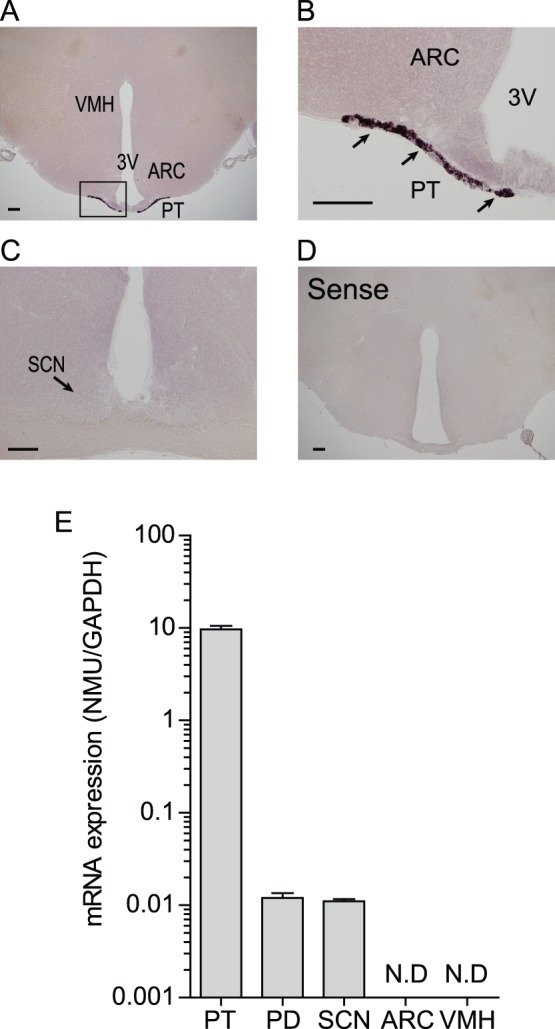
NMU mRNA expression regions in the brain of male Wistar rats. (A) Microphotograph of NMU mRNA expression regions detected by ISH staining on free-floating brain sections with DIG-labeled cRNA probes for rat whole brain. A strong signal was detected in the PT. No signal was detected in the other areas, including the VMH and ARC. (B) Magnified image of the boxed area in A. NMU mRNA-expressing cells were widely distributed throughout the PT. (C) No signal for NMU mRNA expression was detected in the SCN. (D) Sense RNA probes to NMU generated no specific signal. Scale bar: 200 µm. PT, pars tuberalis; ARC, arcuate nucleus; VMH, ventromedial hypothalamic nucleus; 3V, third ventricle; SCN, suprachiasmatic nucleus. (E) qPCR analysis of NMU in the PT, PD, SCN, ARC, and VMH using LMD samples. High expression was detected in the PT, low expression was detected in the PD and SCN, and no expression was detected in the ARC and VMH. All values are means ± S.E.M. (n = 4).

To determine NMU mRNA expression regions in the rat brain, we performed ISH on free-floating sections with DIG-labeled cRNA probes for rat whole brain, including the medulla oblongata. Strong staining for NMU mRNA was observed in the PT, and NMU mRNA-expressing cells were widely distributed throughout the PT (Fig. 1A, B). On the other hand, no signal was detected in any other regions, including the VMH, ARC, and SCN, where NMU mRNA expression has been previously reported in the rat brain using radioisotope-labeled probe [16], [30] (Fig. 1A, C). Sense RNA probes to NMU mRNA generated no specific signal (Fig. 1D). In addition, qPCR was also performed using LMD samples to compare the expression level of NMU mRNA among the PT, PD, SCN, ARC, and VMH. We found that NMU mRNA expression in the PT is approximately 1000 times higher than that in the PD and SCN (Fig. 1E). No mRNA expression was found in the ARC or VMH (Fig. 1E).

### NMU mRNA-expressing Cell Types in the PT

**Figure 2:**
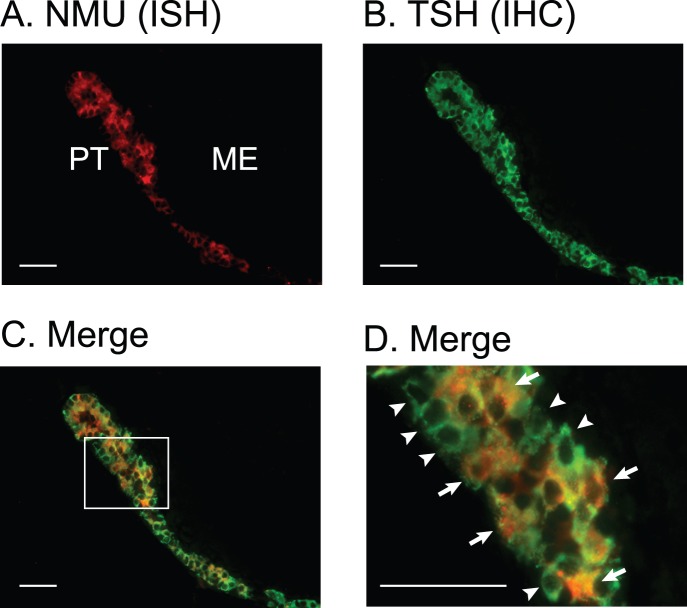
NMU mRNA-expressing cell types in the PT. Microphotograph of NMU mRNA expression detected by double staining by HNPP fluorescent ISH for NMU mRNA and by fluorescent IHC for TSH in fixed, frozen 5-µm sections. (A) Microphotograph of NMU mRNA expression detected by HNPP fluorescent ISH (red). NMU mRNA-expressing cells were widely distributed in the PT. (B) Microphotograph of TSH detected by fluorescent IHC staining. Many TSH-immunostained cells were distributed throughout the PT (green). (C) Microphotograph of merged pictures of double staining for NMU mRNA expression, which was detected by HNPP fluorescent ISH and TSH detected by fluorescent IHC. Yellow color represents the merged signal for NMU mRNA and TSH production. Although almost all NMU-expressing cells showed TSH immunoreactivity (arrows, yellow), cells exhibiting only TSH immunostaining were also found (arrowheads, green). (D) Magnified image of the boxed area in C. NMU mRNA-expressing and TSH producing cells (arrows, yellow) and cells producing only TSH (arrowheads, green) exist. Scale bars: 50 µm. PT, pars tuberalis; ME, median eminence.

To determine the NMU mRNA-expressing cells in the PT, we performed double staining by HNPP-fluorescent ISH for NMU mRNA and fluorescent IHC for TSH (Fig. 2). Both NMU mRNA-expressing cells (Fig. 2A, red) and TSH immunostained cells (Fig. 2B, green) were distributed throughout the PT. Although almost all of the NMU-expressing cells also showed TSH immunoreactivity (Fig. 2C, D, yellow), cells that were immunostained only for TSH were also detected (Fig. 2C, D, green).

### Diurnal Change of NMU mRNA Expression in the PT

**Figure 3:**
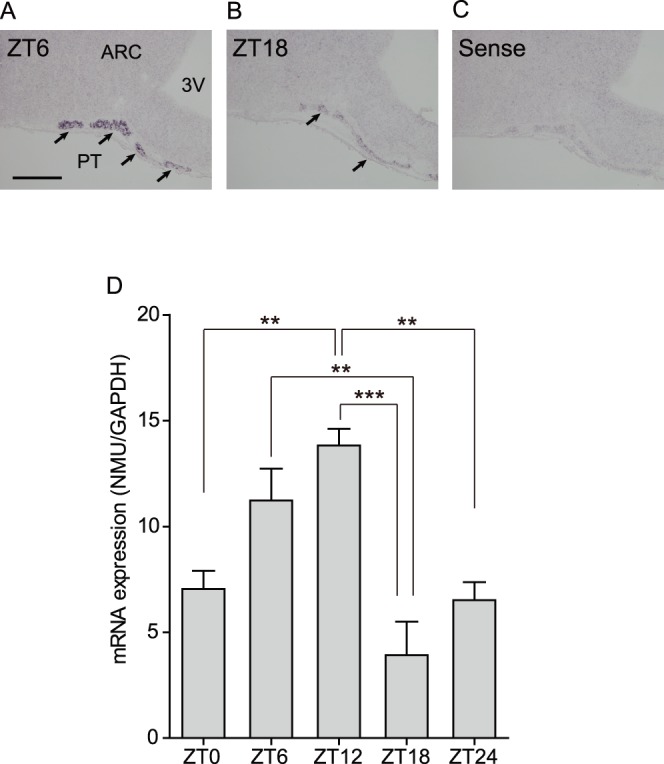
Diurnal change of NMU mRNA expression in the rat PT. (A, B) Microphotograph of NMU mRNA expression detected by ISH staining on fresh frozen sections. The staining signal in the PT (arrows) was high at ZT6 (light phase) and low at ZT18 (dark phase). (C) Sense RNA probes for NMU generated no specific signal. Scale bar: 200 µm. PT, pars tuberalis; ARC, arcuate nucleus; 3V, third ventricle. (D) qPCR analysis of NMU using LMD samples of the PT at ZT0, 6, 12, 18, and 24. The expression level fluctuated in a time-dependent manner. NMU mRNA expression was low at ZT0 and gradually increased, and then reached a peak at ZT12. The level was decreased at ZT18 and remained low at ZT24. All values are means ± S.E.M. (n = 4). Significant differences were detected by one-way analysis of variance (*P = *0.0004) and Tukey’s multiple comparison test (** *P*<0.01, *** *P*<0.001).

Because we previously found that αGSU and TSHβ mRNA expression showed diurnal change in the rat PT [11], we hypothesized that NMU mRNA expression also showed diurnal change. Thus, ISH for NMU was performed on the PT at ZT6 (light phase) and ZT18 (dark phase). The staining signal in the PT was strong at ZT6 and weak at ZT18 (Fig. 3A, B). In addition, these changes in staining properties of serial sections from the rostral to caudal portions of the PT were synchronized throughout the PT (data not shown). Sense RNA probes to NMU mRNA generated no specific signal (Fig. 3C). After these experiments, we analyzed the change of NMU mRNA expression with LMD samples of the PT at ZT0, 6, 12, 18, and 24. The expression level fluctuated significantly in a time-dependent manner. While the NMU mRNA expression level was low at ZT0, it was increased at ZT6 and reached its highest at ZT12. Then, the expression level dramatically decreased at ZT18 and remained low until ZT24 (Fig. 3D).

### Effects of Melatonin on NMU mRNA Expression in the PT, PD, and SCN

**Figure 4:**
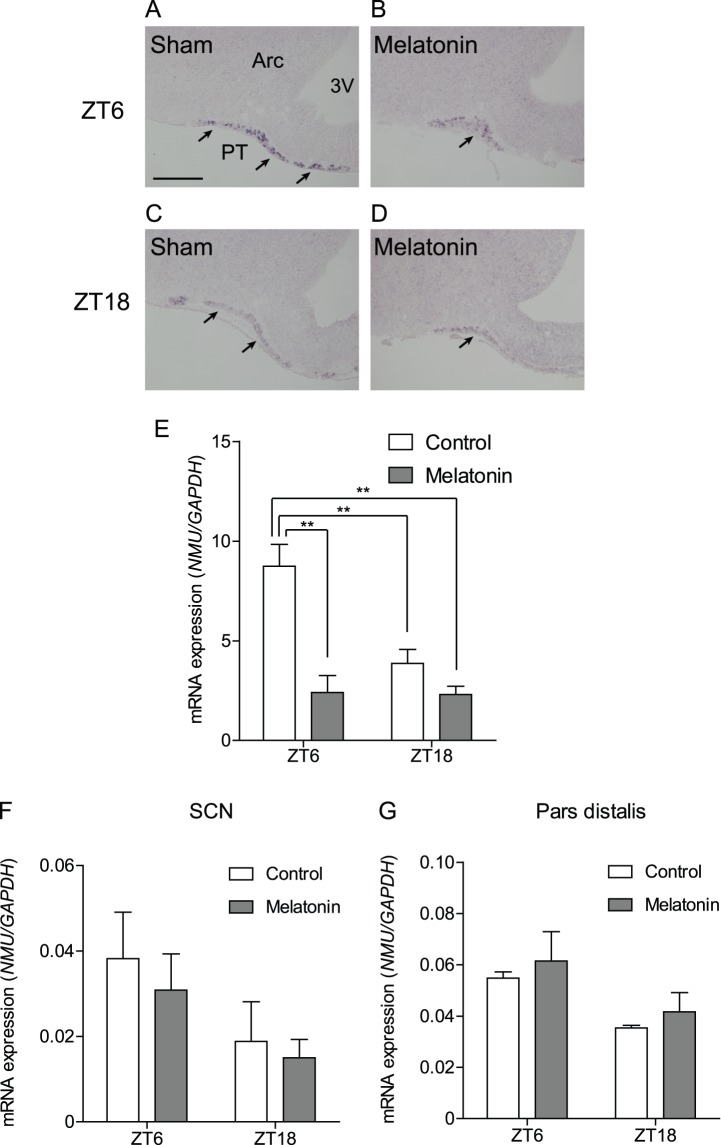
Effects of melatonin on NMU mRNA expression in the PT, PD, and SCN. (A–D) Microphotograph of NMU mRNA expression detected by ISH in the PT of control and melatonin-treated groups at ZT6 and ZT18. (A, B) Staining signal in the PT was decreased in the melatonin-treated group compared to the sham group at ZT6. (C, D) In the ZT18 samples, a similar staining intensity was observed in the sham and melatonin-treated groups. Scale bar: 200 µm. PT, pars tuberalis; ARC, arcuate nucleus; 3V, third ventricle. (E) qPCR analysis of NMU in the PT was performed for the sham and melatonin groups at ZT6 and ZT18 using LMD samples. Two-way ANOVA revealed a significant interaction between melatonin treatment and time point (*P*<0.05) and that the NMU mRNA expression level of the ZT6 melatonin group was significantly higher than that of the ZT6 melatonin, ZT18 control, and ZT18 melatonin groups (one way ANOVA, *P*<0.01). (F, G) qPCR analysis for NMU in the SCN and PD performed for the sham and melatonin-treated rats at ZT6 and ZT18. NMU mRNA expression in the SCN and PD were considerably lower and not influenced by time or melatonin treatment.

Because MT1 is highly expressed in the PT, we next examined the effects of melatonin on NMU mRNA expression in the PT *in vivo*. After chronic melatonin administration for 7 days, ISH studies revealed that immunoreactivity was considerably reduced in the melatonin-treated group compared to the sham group at ZT6 (light phase) (Fig. 4A, B). At ZT18 (dark phase), a similar staining level was found between the sham and melatonin groups (Fig. 4C, D). qPCR analysis of the LMD samples was also performed to clarify the effect of melatonin on NMU mRNA expression levels. A two-way ANOVA revealed the significant main effect of melatonin treatment (*P*<0.01). The interaction between melatonin treatment and time (ZT6 and ZT18) was significant as well (*P*<0.05), and Tukey’s test for multiple comparison between groups revealed that the ZT6 control differed significantly from the ZT18 control, ZT18 melatonin treatment, and ZT6 melatonin treatment groups (*P*<0.01 each) (Fig. 4E).

On the other hand, a comparison of NMU mRNA expression at ZT6 (light phase) and ZT18 (dark phase) in the SCN and PD showed no significant difference. Melatonin treatment also showed no effect on NMU mRNA expression in the PD and SCN (Fig. 4F, G).

### Effects of Fasting on NMU mRNA Expression in the PT

**Figure 5:**
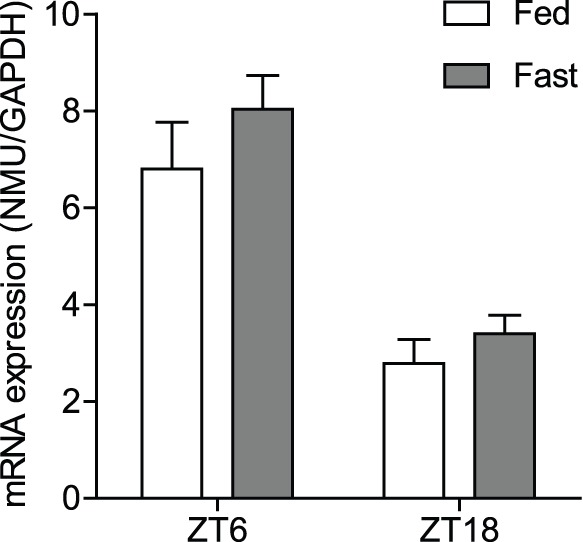
Effects of fasting on NMU mRNA expression in PT. qPCR analysis of NMU in the PT performed for the sham and fasting groups at ZT6 and ZT18. The NMU mRNA expression level was not changed when the sham group was compared with the fasting group at both sampling times, ZT6 (fasting from ZT6 to ZT6 for 48 h) and ZT18 (fasting from ZT18 to ZT18 for 48 h), and the diurnal change was maintained even under fasting conditions (high at ZT6, low at ZT18, *P*<0.01; ZT6 control vs. ZT18 control, ZT6 fasting vs. ZT18 fasting).

We next studied the effects of fasting on NMU mRNA expression in the PT with qPCR, because NMU is known to be involved in the regulation of food intake and energy balance. Regardless of 48 h fasting, the NMU mRNA expression level was not changed compared with the sham group at both sampling times, ZT6 (fasting from ZT6 to ZT6 for 48 h) and ZT18 (fasting from ZT18 to ZT18 for 48 h). The diurnal change of NMU mRNA expression was maintained even under fasting conditions (high at ZT6 and low at ZT18, *P*<0.01; ZT6 control vs. ZT18 control, ZT6 fasting vs. ZT18 fasting) (Fig. 5).

### NMU Receptor type 2 (NMUR2) mRNA Expression in the Rat Brain

**Figure 6:**
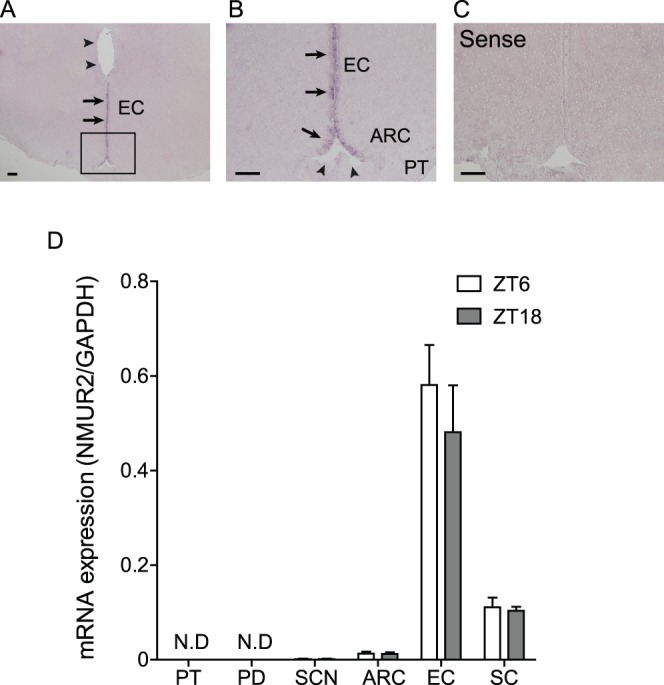
NMU receptor type 2 mRNA expression in the rat brain. (A) Microphotograph of NMU mRNA expression detected by ISH in the fixed frozen sections of the rat brain. Staining signal was detected in the EC of the 3V (arrows). Upper part of the EC of 3V did not show NMUR2 expression (arrowheads). (B) Enlarged view of A. The staining signal was found in the EC of the 3V (arrows), but not in the bottom of the 3V, adjacent to the median eminence (arrowheads). (C) Sense RNA probes to NMUR2 generated no specific signal. Scale bar: 200 µm. PT, pars tuberalis; ARC, arcuate nucleus; 3V, third ventricle; EC, ependymal cell layer of the third cerebroventricle. (D) qPCR analysis of NMUR2 in the PT, PD, SCN, ARC, EC, and SC at ZT6 and ZT18. NMUR2 expression was detected in the EC at the highest level, followed by the ARC and SC, and was detected in the SCN at a considerably lower level. NMUR2 expression was not detected in the PT and PD. NMUR2 expression did not change at either time point in the SCN, ARC, EC, and SC.

ISH for NMUR2 was performed on the rat brain, and a strong staining signal was detected in the ependymal cell layer (EC) of the third cerebroventricle (3V) as previously reported [15] (Fig. 6A, B). However, the upper part of the EC of 3V and the EC at the bottom of the 3V, adjacent to the median eminence, did not show NMUR2 expression (Fig. 6A, B). No specific signal was found in the sense RNA probe reaction (Fig. 6C). qPCR analysis with LMD samples revealed that high and moderate levels of NMUR2 expression were found in the EC and spinal cord (SC), respectively, in comparison with other regions, i.e., the PT, PD, SCN, and ARC. Furthermore, no change in NMUR2 mRNA expression was found between ZT6 and ZT18 in the EC, SC, SCN, and ARC (Fig. 6D).

## Discussion

The PT is thought to be a mediator of seasonal/circadian signals because it expresses a high density of the melatonin receptor. In fact, in birds and seasonal mammals, a relationship between PT and seasonal regulation of GnRH and prolactin secretion has been reported [38], [39]. However, in non-seasonal mammals such as the rat, physiological functions of the PT are not known.

Using ISH on free-floating rat brain sections, we confirmed strong NMU mRNA expression in the rat PT, as previously reported [15], [16]. However, we could not detect any NMU signals in the rat SCN. The strong NMU signals in the rat SCN have been previously shown using ISH with a 35[S]-labeled probe [16] and by Immunohistochemical analysis [40]. The lack of the signals in the SCN in our study could have occurred due to the low sensitivity of detection of our non-RI ISH using the DIG-labeled cRNA probe since we successfully detected the signal in the SCN by qPCR using the LMD sample. Taking together the results of our ISH study and the qPCR analysis, the PT is the highest NMU-expressing region in the brains of the male Wistar rats used in our study.

In this study, we found that NMU mRNA expression in the rat PT has a diurnal change, i.e., high level in the light phase and low level in the dark phase. Furthermore, melatonin treatment significantly inhibited *in vivo* NMU mRNA expression in the PT at ZT6 and slightly inhibited expression at ZT18. It is also known that αGSU and TSHβ mRNA expression in the PT exhibits diurnal changes, and their expression is inhibited by melatonin administration *in vivo* [11]. We previously showed the rhythm of plasma melatonin concentration in Wistar rats. The concentration was considerably low during the light phase (ZT0 to ZT12) and then increased at the beginning of the dark phase, reaching a maximum level at ZT20. After that, the concentration decreased at ZT24 [11], indicating that this circadian rhythm is inversely correlated with the diurnal change of NMU mRNA expression. In this study, we showed that NMU mRNA is expressed in TSH cells and not in other PT cells, suggesting that NMU-expressing cells have MT1 because MT1 only exists in the TSH cells of the rat PT [5]. Hence, melatonin could directly affect NMU mRNA-expressing cells, similar to TSH cells. Several studies have demonstrated that melatonin downregulates the phosphorylation of cAMP response element (CRE)-binding protein in PT cells by inhibiting the activity of adenylyl cyclase through MT1 [41], [42]. Moreover, it has been reported that CRE-like elements are present in the promoter regions of TSHβ and αGSU genes [43], [44], suggesting that intrinsic melatonin suppresses TSH gene expression in PT-TSH cells through these signaling pathways. Although the promoter activity of NMU has not been reported, it is thought that NMU mRNA expression is regulated by melatonin through the same signaling pathway. Taken together, it is strongly suggested that, under normal physiological conditions *in vivo*, endogenous melatonin suppresses NMU mRNA expression during the dark phase and, thus, NMU mRNA expression in the PT has a circadian rhythm. However, our results cannot completely separate the effects of melatonin and the effects of circadian rhythmicity. If NMU in the PT is regulated primarily by melatonin, we predict that pinealectomized rats will have chronically elevated NMU expression, and that acute melatonin treatment will reduce these levels. On the other hand, a rhythm in NMU expression in the PT of pinealectomized rats would suggest an additional circadian mechanism for regulation of NMU.

Because of the lack of specific antibodies for rat NMU, we could not study NMU production and secretion in the PT at this time. We previously studied the ultrastructural features of PT-TSH cells and reported that the cells have a well-developed golgi apparatus, a rough endoplasmic reticulum, and few secretory granules, suggesting that these cells are in a hyperfunctional state and secrete TSH in a constitutive manner [2], [45]. In fact, we confirmed that PT-TSH was secreted in a constitutive manner, without accumulating in the cells, in the slice culture experiment [37]. Because NMU is also produced in PT-TSH cells, NMU might be secreted with constitutive pathways similar to PT-TSH, and NMU secretion from the PT could have a circadian rhythm according to its mRNA expression.

Although NMU in the rat PT seems abundant, its physiological function remains to be clarified. It is well known that central NMU is involved in the regulation of food intake and energy balance. In the rat, a single icv injection of NMU gives a clear catabolic response; it increases core body temperature and locomotor activity and reduces food intake [15], [30], [31], [36]. From these and other studies, high concentrations of NMU in the cerebroventricular fluid can suppress feeding and activate multiple regions of the brain. As also shown in this study, NMUR2 is highly expressed in the EC of the third cerebroventricle (3V), and it has been suggested that some of these responses are likely transduced through NMUR2 in the EC of 3V [15]. However, the origin/sources of NMU remain unclear [16]. NMU is a brain-gut hormone that has been detected in numerous peripheral tissues, including the stomach and intestine of humans and rats [25], [46], [47]. Therefore, it is of concern whether peripheral NMU can gain access to the brain [16]. Although this point is not yet clear, some reports have shown that peripheral injection of NMU does not influence food intake or core body temperature [47], suggesting that the effects induced by icv injection of NMU could be caused by centrally produced NMU. The PT is anatomically located at the base of the brain and is bathed in cerebroventricular fluid. Aguado LI et al. and M. Guerra et al. have studied in detail the structure of the rat PT and showed that the PT possesses intercellular channels in open communication with the subarachnoid space, with no barrier between the subarachnoid space and the PT. Furthermore, they have reported that the subarachnoid space may be regarded as a probable route for the transport of trophic factor(s) and/or secretory product(s) of the PT, suggesting all PT cells are directly exposed to the cerebroventricular fluid and that PT secretory cells have a unique secretory pathway [48], [49]. Taken together, NMU of the PT could be secreted into the cerebroventricular fluid, and the PT may be the origin of the high concentration of NMU in the cerebroventricular fluid. In fact, it has been hypothesized that TSH of the PT also retrogradely affects the TSH receptor expressed in the EC of the third cerebroventricle (3V), through the vascular system, extracellular space, or tanycyte processes [50]. Although it must be elucidated in future studies, the PT might send its signals including TSH and NMU to the hypothalamus, especially the EC of the 3V in the retrograde pathway, not only to the PD through the hypophyseal portal system in anterograde pathway.

A few reports have demonstrated a link between energy balance and NMU of the PT. Nogueiras R et al. have shown that NMU mRNA expression in the rat PT was diminished by leptin icv injection, despite the fact that the leptin receptor does not exist in the PT [51]. Ivanov TR et al. reported decreases in NMU expression in the PT of obese (fa/fa) Zucker rats [15]. They also observed a decrease in NMU mRNA expression in the PT of 48-h fasted rats, suggesting that NMU mRNA expression in rat PT is downregulated by negative energy states, which may be mediated indirectly by changes in leptin levels. However, in our present study, 48-h fasting exposure did not affect NMU mRNA expression, as NMU mRNA expression consistently showed diurnal rhythm, high during the light phase and low during the dark phase. Although there is no explanation for this discrepancy, our result suggests that NMU mRNA expression in the PT is not influenced by a negative energy state and it retains the diurnal change, which depends on the external environment state of a light-dark cycle. It is thought that NMU of the PT relates to the basic feeding activity of nocturnal animals, such as eating food mostly at night.

In this study, we showed that NMU mRNA in the PT was the highest in the rat brain and that all NMU-expressing cells in the PT produce TSH. Its expression has a diurnal change that is high during the light phase and low during the dark phase, and this is likely regulated by melatonin level and the environmental light-dark cycle. Although further studies are needed, NMU produced in the PT might be secreted into the cerebroventricular fluid where it can act on the NMUR2 of the EC of 3V.
